# Implementation of N-Interval fourier transform analysis - Application to compound action potentials

**DOI:** 10.1016/j.mex.2023.102441

**Published:** 2023-10-21

**Authors:** G. Fischer, M. Kofler, D. Baumgarten

**Affiliations:** a Institute of Electrical and Biomedical Engineering, UMIT – Private University for Health Sciences and Health Technology, Eduard Wallnoefer Zentrum 1, 6060 Hall in Tirol, Austria; b Department of Neurology, Hochzirl Hospital, 6170 Zirl, Austria; c Institute of Biomedical Engineering and Informatics, Technische Universitt Ilmenau, G.-Kirchhoff-Str. 2, 98693 Ilmenau, Germany

**Keywords:** Somatosensory evoked potentials, Spectral analysis, Waveform averaging, N-Interval Fourier Transform Analysis (N-FTA)

## Abstract

N-Interval Fourier Transform Analysis (N-FTA) allows for spectral separation of a periodic target signal from uncorrelated background interference. A N-FTA pseudo-code is presented. The spectral resolution is defined by the repetition rate of the near periodic signal. Acceptance criteria for spectral targets were defined such that the probability of accepting false positives is less than $\frac{1}{500}$. Simulated and recorded neural compound action potentials (CAPs) were investigated. Simulated data allowed for comparison with reference solutions demonstrating the stability of N-FTA at conditions being comparable to real world data. Background activity was assessed with small errors. Evoked target components were assessed down to power spectral density being approximately N times below the background level. Validation was completed investigating a measured CAP. In neurophysiological recordings, this approach allows for accurate separation of near periodic evoked activity from uncorrelated background activities for frequencies below 1kHz.

• *N*-FTA allows for spectral separation of a periodic target signal from uncorrelated interference by analyzing a segment containing *N* target signal repetitions.
• A MATLAB implementation of the algorithm is provided along with simulated and recorded data.
• *N*-FTA was successfully validated using simulated and measured data for CAPs.

## Graphical abstract

**Figure:**
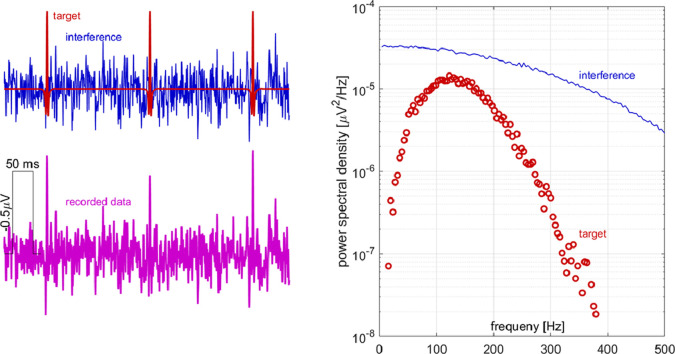

Specifications Table

**Table:** 

Subject area:	Signal Processing
More specific subject area:	Spectral Analysis
Name of your method:	N-Interval Fourier Transform Analysis (N-FTA)
Name and reference of original method:	G. Fischer, J. Haueisen, D. Baumgarten, M. Kofler.
	Spectral separation of evoked
	and spontaneous cortical activity
	- Part 1: Delta to high gamma band.
	Biomedical Signal Processing and Control,
	submitted, 2023.
Resource availability:	Data and source code can be downloaded using the link below.
MATLAB codes and data are available at	umit-tirol.at/iebe
	see also Table 1.

## Methods details

The scope of this section is to provide a detailed implementation of N-FTA [1] in a theoretically sound context. Somatosensory evoked potentials (SEPs) are a well established diagnostic tool extracting target signals from uncorrelated background activity by repeated stimulation. Compound Action Potentials (CAPs) are a type of evoked potential recorded from longitudinal neural tracts. The chosen terminology links actual implementation to SEPs [3], [9]. The first subsection defines the underlying assumptions. The next two subsections describe implementation and necessary details for obtaining sufficient accuracy. The last subsection provides a validation of the method. Additional information about the context of this research is provided in a supplement.

**Table 1:** Test signals for download.

filename	description
StSignal_CAPsim_010nV_WN.mat	synthetic CAP with white noise at low level
StSignal_CAPsim_100nV_WN.mat	synthetic CAP with white noise at high level
StSignal_CAPsim_050us_JIT.mat	synthetic CAP with jitter at low level
StSignal_CAPsim_500us_JIT.mat	synthetic CAP with jitter at high level
StSignal_CAPsim_TimeDrift.mat	synthetic CAP with activation drift
StSignal_CAPsim_AmplitudeVar.mat	synthetic CAP with amplitude variation
StSignal_CAPsim_AllVariations.mat	synthetic CAP investigated in [1].
StSignal_CAPju9_C395.mat	human CAP investigated in section 3.3.
StSignal_MED47e_C395.mat	cortical signal of subject B in [1].

### Finite periodic signal – Definition

**Fig. 1:**
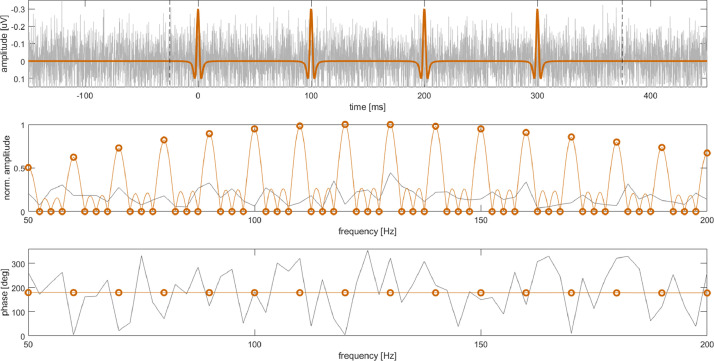
*Top panel:* Finite periodic signal with N=4 repetitions (orange) at $f_{E}=\text{10}\text{Hz}$ and stochastic white noise (gray). The vertical dashed lines mark the finite periodic interval. According to clinical practice, negativity was plotted upwards. The first peak was centered at time point zero. *Middle panel:* Time-continuous FT of the finite periodic signal (narrow orange line) and its N-FT (orange circles) within a frequency interval around the maximum. White noise displays random variations in the spectrum (gray trace). *Bottom panel:* For the finite periodic signal phase was plotted for multiples of $f_{E}$ (orange markers). For white noise phase was stochastic (gray). The same frequency interval as in the middle panel was used..

As a basic model, repetitive evoked recordings may be approximated by a series of N identical responses which are corrupted by irregular background activity [7]. Fig. 1 depicts an example for illustration. Here, the evoked signal contained N=4 identical simulated CAPs, as obtained from [2]). They were repeated at a stimulation interval $T_{E}$ of 100ms (i.e. 10Hz stimulation rate or evoked frequency $f_{E}$). White noise with a standard deviation of 0.1μV was used as a model for irregular background activity.

A signal containing N identical repetitions is a finite periodic signal. In a strict mathematical terminology it is **a-periodic** as it contains two isoelectric segments of infinite duration. Writing $F\{\phi_{1}\}$ for the *time-continuous* Fourier transform (FT) of a single response $\phi_{1}$, the FT of a series $\phi_{N}$ of N identical responses has been obtained from [7]

(1) $$F\left\{\phi_{N}\right\}=F\left\{\phi_{1}\right\}\frac{1}{N}\frac{1-e^{-iN\frac{f}{f_{E}}}}{1-e^{-i\frac{f}{f_{E}}}}.$$

Fig. 1 shows the magnitude of the *time-continuous* Fourier transform (FT) of the exemplary finite periodic signal. FT showed local maxima at multiples $kf_{E}$ (i.e. at the evoked harmonics) and regularly spaced zero crossings in between the evoked harmonics. This pattern was governed by Eq. (1). Experimental data requires the investigation of a finite time interval. For convenience we chose exactly the finite periodic interval. Generally, DFT assumes that the signal was periodically repeated outside of the finite periodic interval. Thus, we obtained a **periodic** signal with a fundamental frequency $f_{E}$. The discrete frequency samples were exactly at the evoked harmonics or at the zero crossings (Fig. 1). A frequency resolution of $\frac{f_{E}}{N}$ was obtained. We refer to the DFT of a finite periodic interval by the term N-FT.

As can be observed from Fig. 1, the N-FT of the finite periodic evoked signals contained non-zero components only at the evoked harmonics. An irregular background signal contained non-zero elements at all frequencies. We summarize therefore:

• Harmonic components $kf_{E}$ represent a superposition of the evoked signal and irregular background activity.
• Non-harmonic components represent the spectrum of irregular signals, such as noise or physiological background activity.

We investigated also phase in the spectrum. As it follows from the time shift theorem of Fourier transform, actual phase depends on the definition of the time point zero. For the example depicted in Fig. 1 we chose time zero at the first peak in the signal. For the almost even shape of a CAP pulse in the example, the N-FT at the evoked harmonics contained essentially cosine terms with a negative sign. Thus, phase was almost constant and near $180^{\circ }$. To our experience, visual interpretation of phase plots is simplified by selecting time zero near an early morphological marker in the signal. Therefore, we have included the option of selecting time zero in our implementation. However, our implementation of N-FTA does not necessarily require this selection.

### N-FTA – Pseudocodes

*Numerical value of the evoked frequency.* In our experimental recordings containing some 100s of data at a sample rate near 10kHz, the number of data points M in the interval exceeded a million. For accurately capturing the evoked harmonics in the spectrum the evoked frequency must be numerically represented with an accuracy in the ppm (parts per million) range. Note that for state of the art electronic devices, the uncertainty in stimulation frequency or sample rate is in the order of some ppm.

In our software implementation, we chose the following approach for obtaining a sufficiently accurate numerical value for the evoked rate $f_{E}$. We chose the time frame of data sampling as the reference and set the sample rate $f_{S}=\text{9.6}k\text{Hz}$ (i.e., the nominal sampling rate of the biopotential amplifier, g.USBamp g.tec GmbH, Austria, see [1]). We computed $f_{E}$ based on this reference. In our experimental data the segment after the last stimulus was not complete. Thus, the data contained N+1 stimuli but N usable repetitions. Our code determined M as the number of time steps from the first stimulus to the last time step ahead of stimulus N+1. The evoked frequency was then obtained by:

(2) $$f_{E}=f_{S}\frac{N}{M}.$$

In our experiments stimulation rate was set to 2.46 and 3.95Hz, respectively). The fundamental frequency as computed by N-FTA from experimental data was only about 17ppm below the preset values. Thus, a sufficiently high accuracy was obtained in the experimental setting.

*Pseudocode*N-FTA applies three steps. In a first step (see Algorithm 1) the preprocessed signal is loaded. Here, preprocessing involves removal of stimulation artifacts by subtraction of an artifact template (i.e., the average shape of all stimulation artifacts obtained during a given recording session of 600 or 1000 repetitions, respectively) and the removal of linear drift components (see also [1]). Then, a DFT of the finite periodic interval is performed (N-FT). Then, power spectral density w and phase information $\theta^{\prime }$ are computed (see [1]). Here, the function “CheckPhaseInterval” ensures that angles are within a $360^{\circ }$ interval. A data structure St is constructed containing all harmonic frequencies below the Nyquist frequency. The number of repetitions N is stored in the structure St.

**Algorithm 1:**
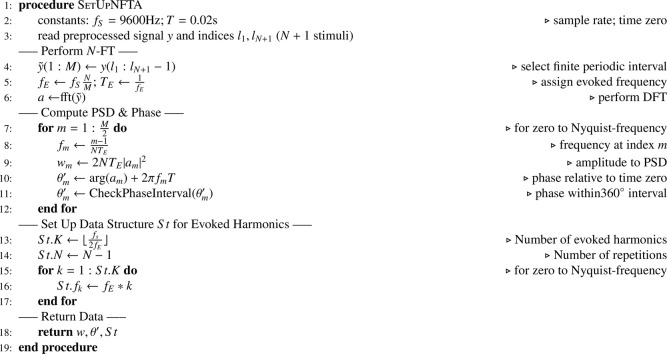
Prepare Data for N-FTA.

In the pseudocodes we use the notation ⌊…⌋ for rounding towards lower integer (“floor”),⌈…⌉ for rounding towards upper integer (“ceil”) and ⌊…⌉ for rounding towards nearest integer (“round”).

In a second step, we assess background activity (see Algorithm 2). Within a loop background activity is computed at each evoked harmonic $kf_{E}$ and the width of the window at each evoked harmonic is slightly below $\pm \frac{f_{E}}{2}$ (“floor” rounding of border indices). The PSD at $kf_{E}$ was always removed from background analysis, as it contains evoked activity. The detection of power line harmonics is based on the observation, that for a powerline harmonic $nf_{PL}$, the “check variable” $f_{check}=kf_{E}-f_{PL}$ must be within the interval $\left[-\frac{f_{E}}{2},\frac{f_{E}}{2}\right]$. A while-loop is used such that $f_{PL}$ is subtracted exactly n-times from $f_{check}$. If the check variable is within the interval, frequencies near the powerline harmonics (interval $\pm \frac{\Delta f_{PL}}{2}$) are removed from the analysis. The average PSD of the data addressed by the indices in the array defined the background level.

**Algorithm 2:**
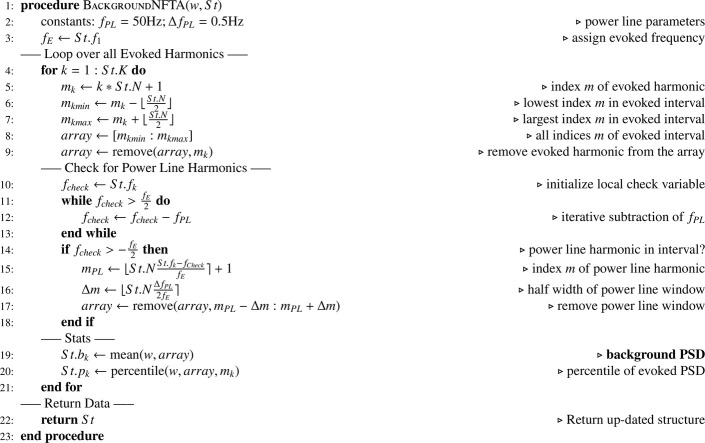
N-FT Background Analysis.

The array is also used for computing the percentile value of the PSD with respect to the distribution of background activity at each evoked frequency. The analysis of evoked frequency components requires a decision about acceptance of each evoked harmonic. This means that evoked components are only considered reliable, if they are relatively large compared to background activity. The pseudocode in Algorithm 3 first initializes the structure field St.A with 0 for all elements. The code then checks for the *Amplitude Phase Acceptance Criterion (APAC) 1*. Upon acceptance of an individual harmonic, the value gets updated to 1. APAC 2 investigates evoked harmonics together with their left and right neighbors. If all three harmonics in such a group were larger than $p_{L}$, a phase check (as described in [1]) is performed. Here, routine CheckPhase selects a proper $360^{\circ }$ interval such that no phase steps occur between neighboring harmonics. Upon acceptance, St.A is set to 1 for all three elements of the group. Finally, the PSD $St.e_{k}$ is computed for all accepted harmonics and normalized to the level of a single repetition. For harmonics, which did not pass the acceptance criteria, the numeric data type NaN (not a number) is assigned to $St.e_{k}$.

**Algorithm 3:**
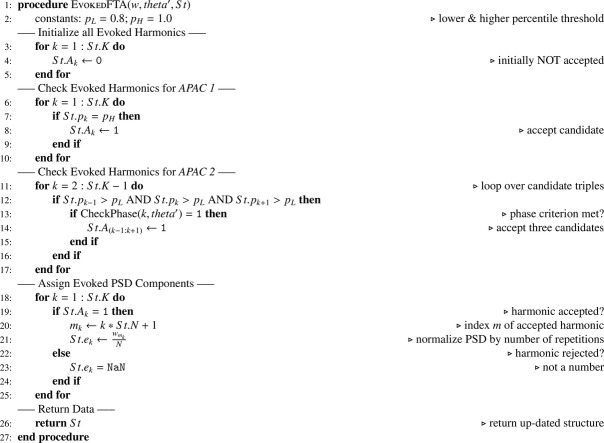
N-FT Evoked Analysis.

As the result of the N-FTA, the structure St contains the evoked frequencies $kf_{E}$ together with the associated background and evoked PSD levels $b_{k},e_{k}$. The evoked frequency $f_{E}$ defines the frequency resolution of the method. We included only frequencies up to 750Hz into the analysis.

### MATLAB-implementation

Along with this communication we provide a MATLAB-implementation for download. This implementation involves three functions reflecting Algorithms 1 to 3 as described above. Furthermore, it involves auxiliary functions such as phase checks. All routines were named such that they can be readily related to the listed pseudocode. MATLAB codes also contain detailed comments.

N-FTA is performed by running the script runNFTA01.m. It prompts for selecting the input signal (provided in data structure StSignal in *.mat format). Next, the script runs the N-FTA, plots the result and assigns results to the data structure StNFTA in the MATLAB-workspace. Furthermore, also PSD and phase are assigned to data structure StDFT for analysis. All data structures are described in detail by the comments within the codes.

### Method validation

This subsection first provides a verification of the accuracy of the implemented code by means of an example. Next, it investigates the influence on variations in individual responses by reviewing theory and by studying numerical examples. Finally, it illustrates application to a human CAP. *Finite Periodic Test* We created a numerical example to test function and accuracy of the implemented code. Chosen parameters matched the values of the experimental setting, i.e., N=1000, $f_{E}=\text{3.95}\text{Hz}$ and $f_{S}=\text{9.6}k\text{Hz}$. As a template for a single response, a simulated CAP was used. The data was taken from a previous study [2] (simulation parameters: conduction velocity $v=60ms^{-1}$, depth of the neural tract s=50mm and temporal dispersion τ=1.25ms). This signal and its amplitude spectrum are depicted in Fig. 1. Its peak-to-peak amplitude was 0.32μV. The original simulation was digitized at a sampling interval of 5μs. For verification purposes the data was down-sampled to $f_{S}=\text{9.6}k\text{Hz}$ within an interval of length $T_{E}$. For creating a finite periodic evoked CAP this segment was repeated N times. The reference PSD for the evoked CAP was obtained by performing a standard DFT for the template of a single response and converting the result to a PSD representation.

For test purposes the signal was corrupted with two levels $\sigma_{W}$ of white noise: 0.01μV and 0.1μV, respectively. For each noise level $\sigma_{W}$ the corresponding background PSD $b_{W}$ was obtained from

(3) $$b_{W}=\sigma_{W}^{2}\frac{2}{f_{S}}.$$

Thus, the two investigated noise levels corresponded to spectral background levels of -56.8dB and -76.8dB, respectively (relative to the $1\mu V^{2}/\text{Hz}$ level).

**Fig. 2:**
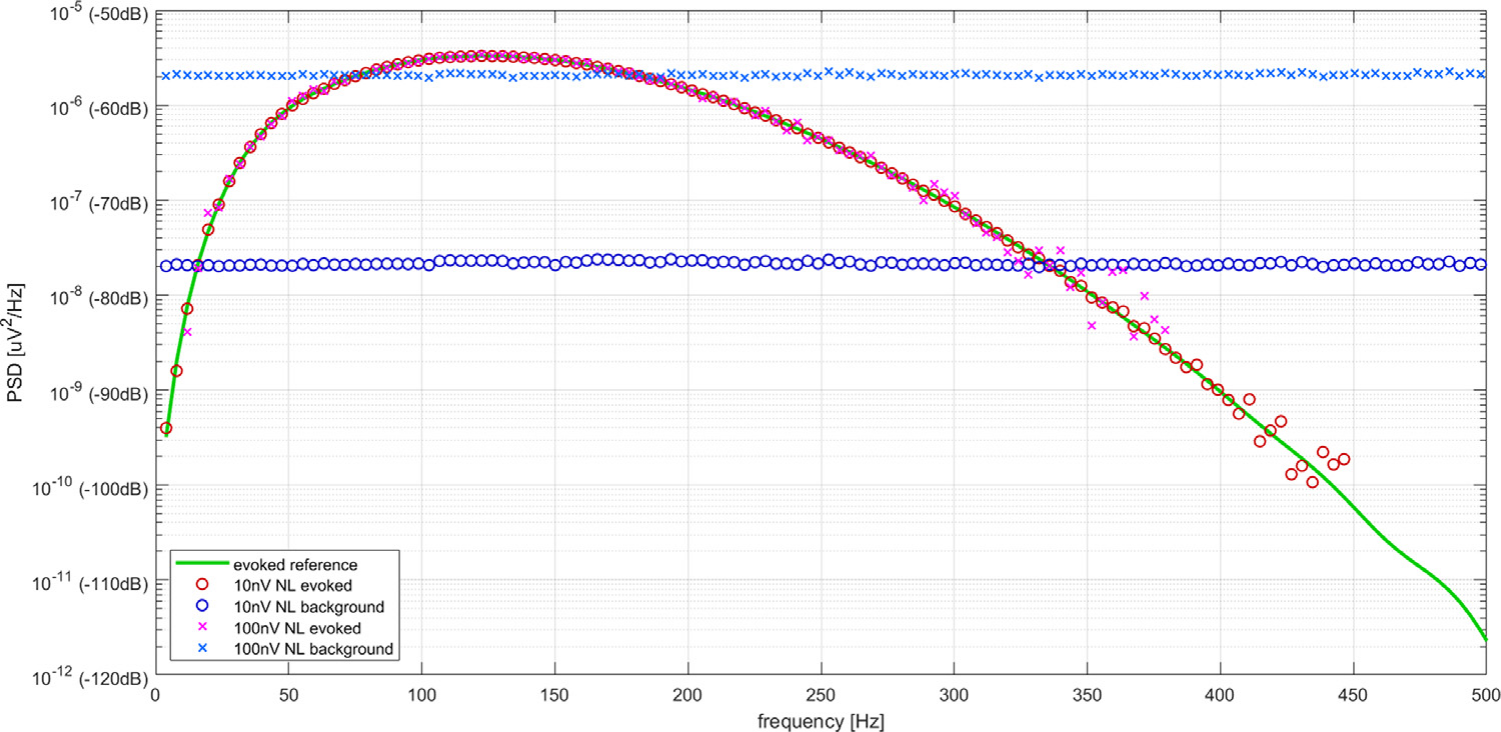
Verification of N-FTA at two noise levels (0.01μV and 0.1μV) by comparison with a reference evoked activity (see text)..

Fig. 2 depicts the reference PSD trace together with the evoked and background components obtained by N-FTA at both noise levels. The two computed background levels match the predicted levels with a residual deviation of 0.00dB ± 0.13dB (mean ± standard deviation) and 0.09dB ± 0.18dB at noise levels of -56.8dB and -76.8dB, respectively. Note that the standard deviation reflects the remaining random variation for the individual white noise pattern. Consistent with the findings from [2] the evoked spectrum is of a band-type with a peak at 122.5Hz. For both noise levels the computed evoked levels match the reference well within the frequency band from 50Hz to 260Hz. Out of this band small but visible deviations were observed. For the higher noise level the evoked frequency was detected from 12Hz to 380Hz. For the lower noise level the interval of detected evoked activity was 4Hzto 446Hz. Evoked activity was detected down to approximately 30dB below the noise level. The errors for the detected evoked components were 1.00dB ± 1.11dB and 0.05dB ± 0.69dB for the high and low noise levels, respectively.

Thus, we verified that N-FTA detects and separates background and evoked activities. The limit for detecting evoked activity is approximately $10\log_{10}\left(N\right)$ [dB] below the background level. The accuracy of the detected evoked components improves with increasing evoked-to-background ratio.

*Variations in Repetitions.* As outlined above, N-FTA assumes that all repetitions were identical. However, in real world experimental settings (small) variations may be observed between individual responses. The scope of this section is to investigate the influence of inter-cycle variations on the accuracy of N-FTA. We investigated three different types of variations and quantify their effect.

**Fig. 3:**
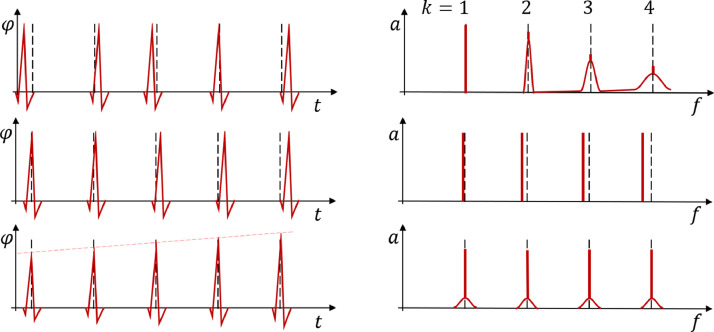
Different types of variations in repeatable signals and their influence on the spectral profile. *Top left:* Irregular activation occurs with a random time shift from a regular action timing (indicated by hashed lines). *Top right:* In the frequency domain irregular activation is reflected by broadening of harmonic peaks with increasing index k. *Middle left:* Activation drift is reflected by a systematic shift in activation timing increasing in magnitude with each repetition. *Middle right:* In the frequency domain activation drift is reflected by a systematic displacement of the harmonic peaks. The magnitude of the displacement increases with index k and its sign is opposite to the time shift. *Bottom left:* A variation in amplitude is reflected by a change of amplitude over time. *Bottom right:* In the frequency spectrum variation of amplitude is reflected by side bands adjacent to each harmonic peak. The peak frequency remains unaffected.

**Irregular Activation / Jitter:** Random variation of the activation threshold causes irregular activation (or activation jitter). Rompelman and Ros investigated irregular activation in part two of their review [8]. The upper row in Fig. 3 provides a qualitative illustration of their findings. Jitter goes along with a broadening of harmonic peaks at higher frequencies. The work of Rompelman and Ros provides an analytic treatment, which allows for an estimation of the Fourier transform for a given statistical jitter distribution δ(T). Here, the Fourier transform of the distribution function F{δ(T)}, i.e., the harmonic function of δ(T), enters the calculation.

As can be seen from Fig. 3 activation jitter has less influence on spectral components of lower frequency. We aim to simplify the analytic expressions listed in [8] such, that a border frequency $f_{B}$ can be estimated, below which jitter has only a minor influence. For the ideal case of regular activity the distribution δ(T) becomes a Dirac pulse and its harmonic function is a constant in the frequency domain. As we have shown in the Appendix of [2] the harmonic function of any distribution function is of a “low pass type”, i.e., it is approximately constant at low frequency and approaches zero at higher frequency.

For a normal distribution $\delta_{n}\left(T\right)$ with standard deviation τ, the harmonic function is a Gaussian curve with a standard deviation of $\frac{1}{2\pi \tau }$. For estimation we assumed that for frequencies below half the standard deviation τ the harmonic function was sufficiently close to a constant value and obtained

(4) $$f_{B}≃\frac{1}{4\pi \tau }.$$

As it was shown in the Appendix of [2], Eq. (4) provides a reasonable estimate also for rectangular or triangular distributions. For verifying Eq. (4) we modified the CAP test signal such that peak latency was randomly displaced with statistic distribution $\delta_{n}\left(T\right)$. We set τ to two test values (i.e. 50μs and 500μs, corresponding to the border frequencies $f_{B}$ 1.6kHz and 160Hz, respectively). Since the original CAP template was digitized at 5μs, we were able to perform accurate time shifts by applying linear interpolation between data points. For studying only the effect of activation jitter, we added no background noise to the CAP signal.

**Fig. 4:**
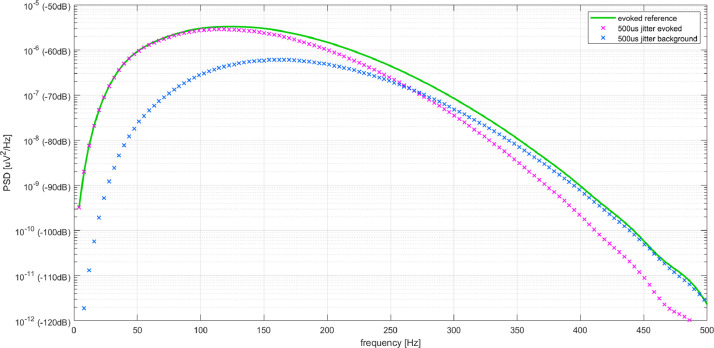
Application of N-FTA to a test signal with activation jitter. An unrealistically high jitter level of 500μs was chosen for illustration (see text).

At the low jitter level (τ=50μs, $f_{B}=\text{1.6}k\text{Hz}$) evoked activity was accurately captured as reflected by an error of -0.05dB ± 0.05dB below 500Hz. At the high jitter level (τ=500μs, $f_{B}=\text{160}\text{Hz}$) large errors were observed at some 100Hz (see Fig. 4). The distortion of the signal spectrum by irregular activation generated spectral components, which were reflected as background activity. Below $f_{B}=\text{160}\text{Hz}$ N-FTA captured evoked activity well and background components were well below evoked activity. Above $f_{B}$, the error in the evoked activity increased rapidly. At 450Hz the estimated error in evoked activity was -8dB and spectral power was almost entirely shifted to background activity.

It must be emphasized that such a high jitter level of τ=500μs is far above a realistic value for an experimental setting, since this would induce time shifts of more than 1ms. This high level was assigned to the synthetic data for illustration purposes. The lower jitter level of τ=50μs appears to be more realistic. In our experimental setting, the width of the stimulation pulse was 200μs. Assuming a rectangular distribution of this width, we obtain a standard deviation of 58μs (see Appendix of [2], equation (32)). Thus, our analysis shows that for frequencies below 1kHz irregular activation or activation jitter may have minor impact on the results, when investigating experimental data.

**Activation Drift:** Physiological effects like, e.g., a drift in body temperature, may cause a drift in the activation timing [5]. As a basic model, a constant drift of activation timing was considered. As it can be seen from Fig. 3, this shifts harmonic peaks. We denote the total time shift occurring over N repetitions by ΔT and obtain for the change $\Delta f_{E}$ of the evoked frequency

(5) $$f_{E}-\Delta f_{E}=\frac{1}{T_{E}+\frac{\Delta T}{N}}≃f_{E}\left(1-f_{E}\frac{\Delta T}{N}\right),$$

yielding

(6) $$\Delta f_{E}=f_{E}^{2}\frac{\Delta T}{N}.$$

We neglect the negative sign, since we are interested in the absolute change in frequency. At each evoked harmonic k, the shift in frequency gets multiplied by k

(7) $$\Delta f_{Ek}=kf_{E}^{2}\frac{\Delta T}{N}.$$

Thus, frequency shifts increase with index k. We aim to estimate a allowable maximal change in frequency $\Delta f_{\max}$ at the highest frequency of interest $f_{\max}=k_{\max}f_{E}$

(8) $$\Delta f_{max}=-f_{max}\Delta T\frac{f_{E}}{N}.$$

The term $\frac{f_{E}}{N}$ defines the frequency resolution obtained for the finite periodic interval. The maximal change in frequency should not exceed half the frequency resolution

(9) $$\Delta f_{max}\leq \frac{f_{E}}{2N}.$$

We obtain by inserting (9) into (8)

(10) $$\Delta T\leq \frac{1}{2f_{max}}.$$

Thus, when aiming to analyze frequencies up to 1kHz, activation drift must be less than 0.5ms. We simulated a time shift ΔT=0.5ms. The errors in the obtained evoked responses were small (-0.19dB ± 0.17dB).

**Variation of Amplitude:** The amplitude of individual responses may vary over time. This can be considered as an amplitude modulation. In the frequency domain, amplitude modulation was reflected by side-bands in each spectral harmonic $f_{En}$ (see Fig. 3).

We simulated such a variation of amplitude by a linear decay from a high initial amplitude to a lower amplitude after N repetitions. We chose a factor of 1.5 at the beginning and a factor of 0.5 after N cycles, thereby investigating a relatively strong variation at an unchanged mean amplitude. The errors in the obtained evoked responses were -0.01dB ± 0.01dB. The irregular activity due to amplitude modulation (side bands) was reflected by a background activity being approximately 10dB below the evoked level.

#### Human CAP

**Fig. 5:**
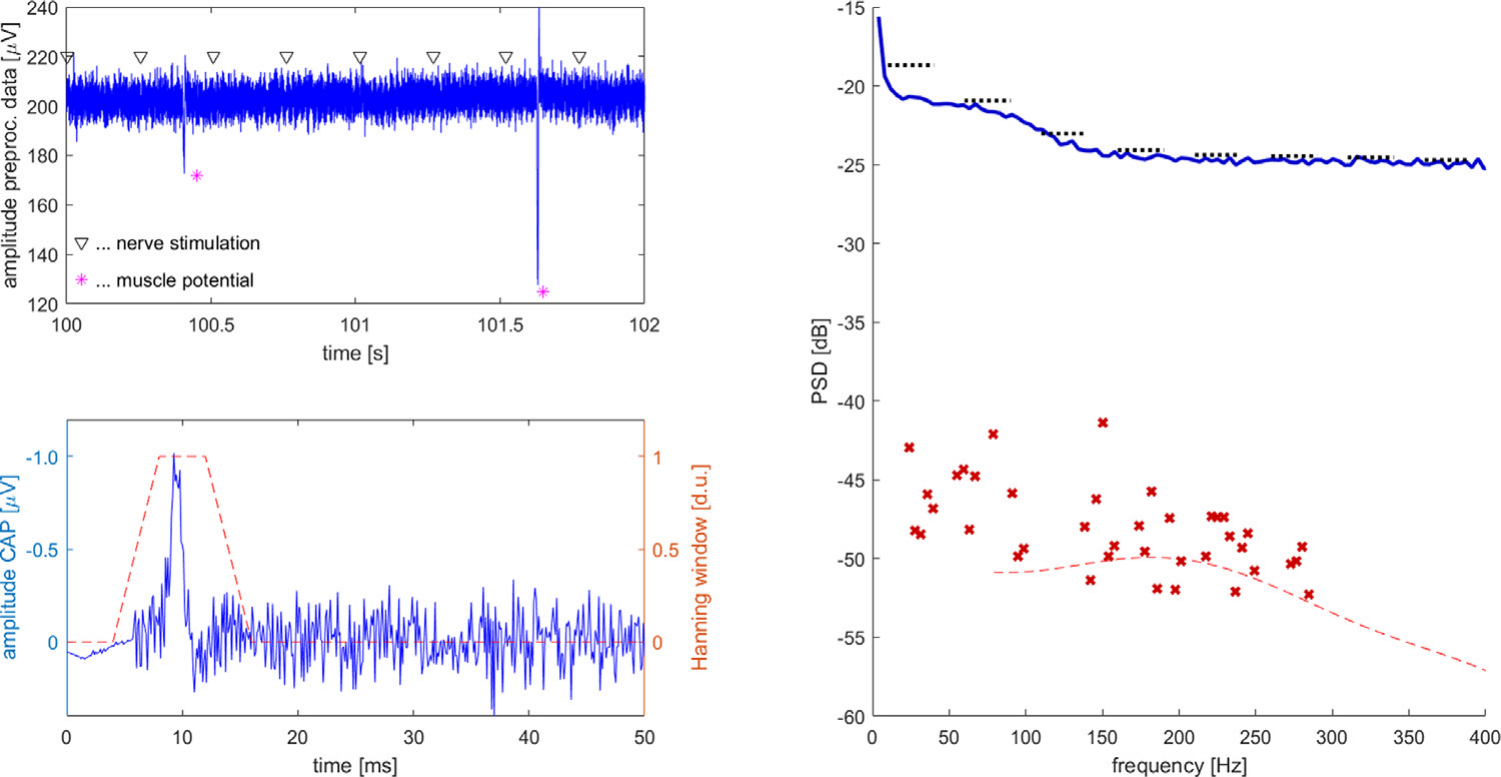
*Top left:* Exemplary segment of preprocessed data. In this segment recorded data displays an offset of approximately 200μV and a background noise floor of approximately 20μV. In addition, two sharp deflections can be observed representing sceletal muscle activity. The triangular markers label the time points of tibial nerve stimulation. The evoked CAP of approximately 1μV amplitude is completely covered by the background noise. *Bottom left:* CAP obtained by averaging 1005 responses. A baseline correction was applied ahead of the onset of the stimulus at time zero. Right after the stimulus a small remaining stimulation artifact can be seen. Neural activation in the popliteal fossa was reflected by a negative peak of approximately 1μV amplitude 9ms after the stimulus (negativity is plotted upwards following clinical convention). Response averaging reduced the noise level to approximately 0.5μV peak-to-peak. *Right:* Background activity as obtained by N-FTA is shown by a blue trace. The dotted lines display estimates computed for verification as described in the text. The evoked activity is displayed by red markers. The Hanning window is displayed in dimensionless units. (For interpretation of the references to colour in this figure legend, the reader is referred to the web version of this article.)

We further applied N-FTA to a CAP recorded in a healthy volunteer. The experimental equipment and data processing was identical as described in [1] and written informed consent was obtained. Briefly, the right tibial nerve was stimulated slightly above motor threshold and a standard bipolar montage was applied at the popliteal fossa [4], [6] for recording the CAP. At an evoked rate of 3.95Hz a segment of approximately 250s was recorded. The finite periodic interval contained slightly more than 1000 repetitions. Data was pre-processed by removing stimulation artifacts and linear drift as described in [1]. Fig. 5 A depicts a segment of 2s duration as an example. The high level of background interference (offset, white noise and muscle potentials) completely masked the evoked CAP. Panel B shows the response averaged CAP as it was obtained from the unfiltered pre-processed data. Peak amplitude was approximately 1μV. Early after the stimulus (below approximately 5ms) a small residual stimulation artifact can be observed after subtraction of a stimulus template.

N-FTA delivers highest background activity at near DC frequencies reflecting remaining nonlinear baseline drift in the pre-processed data. Below approximately 100Hz background interference displays a plateau reflecting muscular activity. At higher frequencies it approaches an approximately constant background level reflecting predominately white noise. The evoked activity was found at comparable PSD-levels and within a similar frequency range as for the synthetic data. Due to the high interference level (about 25dB above the target signal) and the residual stimulation artifacts in the data, the detected components are close to the limit of resolution. This is reflected by a “noisy” distribution of the detected components.

We performed the following analysis for verifying evoked spectral components. For investigating that portion of the averaged signal containing essentially the evoked response, we applied a Hanning-window (corners 4ms, 8ms, 12ms and 16ms, see Fig. 5 B) and computed the power spectral density. In panel C the Hanning-window PSD is depicted for frequencies above 83Hz (the inverse of the total width of the window). It displays a maximum at 182Hz and -50dB. The trace passes within the components detected by the N-FTA.

For verifying background activity, we investigated frequency bands between the harmonics of the powerline frequency, i.e., the bands

(11) $$\Delta_{fk}=\left[\text{10}\text{Hz}\;\text{40}\text{Hz}\right]+k\times \text{50}\text{Hz}\;k=0,1,2,\ldots .$$

For each of these bands we applied a Butterworth-filter of 3^rd^ order to the preprocessed data. We computed the mean squared amplitude for each of these bands and normalized the result by the bandwidth of 30Hz, yielding an estimate for the mean background level in each band. Fig. 5 C displays these estimates deliver similar results as obtained by the N-FTA.

### Validation outcome

A MATLAB-implementation of N-FTA is provided. N-FTA allows for separation of near periodic and uncorrelated background activity in the spectral domain yielding the results in a PSD-representation. Frequency resolution is defined by the fundamental frequency of the periodic component. The ability to separate near periodic spectral components from uncorrelated background activity increases with increasing number of repetitions. As can be taken from [1] the probability for accepting false positive near periodic components is $\leq \frac{1}{500}$ for the current implementation.

Simulated and real repeatedly evoked CAPs were used as test signals. For finite periodic test signals, results matched theoretical predictions with high accuracy. In line with theoretical expectations, increasing level of background noise narrowed the frequency band in which evoked activity was detected. Mathematical treatment and numerical simulations showed that inter-cycle variations in repetitions have stronger influence on the high frequency portion of the spectrum, as compared to low frequencies. This suggest a rather small influence of inter-cycle variations on N-FTA below approximately 1kHz in neurophysiological recordings.

For an example CAP recording (popliteal fossa) the algorithms’ ability for separating evoked and background activity was demonstrated in a relatively broad frequency band at a relatively high level of real-world background activity. As it was further shown in [1], N-FTA allows also for reliable splitting of evoked and background cortical activity. Thus, N-FTA allows for reliable spectral separation of evoked activity from spontaneous background activity within the physiologically relevant frequency range.

## Ethic statements

The experimental data was taken from a study approved by the institutional *Research Committee for Scientific Ethical Questions* (RCSEQ 2632/19). Informed consent was obtained from all subjects.

## CRediT authorship contribution statement

**G. Fischer:** Conceptualization, Methodology, Software, Writing – original draft. **M. Kofler:** Conceptualization, Investigation, Writing – review & editing. **D. Baumgarten:** Resources, Writing – review & editing.

## Declaration of Competing Interest

The authors declare that they have no known competing financial interests or personal relationships that could have appeared to influence the work reported in this paper.

## Data Availability

See above: MATLAB code and data are available at umit-tirol.at/iebe (full link in the specification table)

## Appendix A Supplementary materials

Supplementary Data S1 Supplementary Raw Research Data. This is open data under the CC BY license http://creativecommons.org/licenses/by/4.0/ Supplementary Data S1
