# Supplementary material for: Implementation of N-Interval fourier transform analysis - Application to compound action potentials
Source: MethodsX. 2023 Oct 21;11:102441. doi: 10.1016/j.mex.2023.102441 (PMC10630633; doi:10.1016/j.mex.2023.102441)
Supplement: Supplementary Data S1 — Supplementary Raw Research Data. This is open data under the CC BY license http://creativecommons.org/licenses/by/4.0/ [file mmc1.docx]

**Supplementary Material and Additional Information**

**Supplementary Material**

Simulated and recorded example data sets are provided for download (see Table 1). Furthermore, the MATLAB code is available for download.

**Additional Information and Context**

Signal or waveform averaging is used for recovering repetitive target signals from noisy background activity in a broad spectrum of applications including high speed electronic signals [1] and biosignals [2,7]. Here, signal-to-noise ratio (SNR) is enhanced by ``averaging out" background signals which are stochastic or uncorrelated to the target signal. For further improvement of target recovery, signal averaging can be combined with frequency domain filtering. This requires knowledge about the spectral profile of both target and background components.

In a recent study we focused on separating repeatedly evoked cortical signal components from cortical background activity in the frequency domain [2]. This separation was based on a novel approach named *N*-interval Fourier transform analysis (*N*-FTA). The scope of this *Method X* arcticle is to describe *N*-FTA in methodical detail, to provide a MATLAB implementation of the algorithm to the scientific community, and to provide a validation of results based on simulated and measured data. We have chosen evoked neural compound action potentials (CAPs) [3] as test target signals, since they represent a good model for validating performance.

The obtained solutions provide a direct comparison of evoked and background power spectral density. For the experimental data, background PSD was highest at low frequency reflecting the potential offset due to electrode polarization. Below approximately 150 Hz muscular activity was observable in the background PSD. Above 150 Hz an almost constant PSD level was observed (white noise). The obtained evoked activity was about 25dB below the background activity with highest PSD at a few hundred Hz. It was not detectable above 300 Hz, due to the limited bandwidth of CAPs.

While we used neural signals for introducing this approach, it can be generalized to essentially any superposition of near periodic repetitive signals with uncorrelated interference. Thus, it can be adopted for simultaneous spectral investigation of repetitive signals covered by noise in essentially all fields of digital signal processing.
